# “Microglial nodules” and “newly forming lesions” may be a Janus face of early MS lesions; implications from virus-induced demyelination, the Inside-Out model

**DOI:** 10.1186/s12883-015-0478-y

**Published:** 2015-10-24

**Authors:** Fumitaka Sato, Nicholas E. Martinez, Elaine Cliburn Stewart, Seiichi Omura, J. Steven Alexander, Ikuo Tsunoda

**Affiliations:** Department of Microbiology and Immunology, Louisiana State University Health Sciences Center, 1501 Kings Highway, Shreveport, LA 71130 USA; Center for Molecular and Tumor Virology, Louisiana State University Health Sciences Center, 1501 Kings Highway, Shreveport, LA 71130 USA; Department of Molecular and Cellular Physiology, Louisiana State University Health Sciences Center, 1501 Kings Highway, Shreveport, LA 71130 USA; Department of Neurology, Louisiana State University Health Sciences Center, 1501 Kings Highway, Shreveport, LA 71130 USA

**Keywords:** Preactive multiple sclerosis lesions, Neuropathology, CNS demyelinating diseases, Axonal degeneration, Apoptosis, Oligodendrocytes, Animal models, Experimental autoimmune encephalomyelitis, Theiler’s murine encephalomyelitis virus

## Abstract

**Background:**

Although the precise mechanism of initial lesion development in multiple sclerosis (MS) remains unclear, two different neuropathological findings have been reported as a potential early pathology of MS: “microglial nodules” and “newly forming lesions”, both of which contain neither T cell infiltration nor demyelination. In microglial nodules, damaged axons were associated with a small number of aggregated macrophages/microglia, while oligodendrocyte apoptosis was a characteristic in newly forming lesions. However, is the presence of “microglial nodules” and “oligodendrogliopathy” mutually exclusive? Might these two different observations be the same neuropathology (as proposed by the concept, “preactive lesions”), but interpreted differently based on the different theories of early MS lesion development, using different staining methods?

**Discussion:**

Since two studies are looking at two distinct aspects of early MS pathogenesis (one focused on axons and the other on oligodendrocytes), in a sense, one can say that these two studies are complementary. On the other hand, experimentally, Wallerian degeneration (WD) has been demonstrated to induce both microglial nodules and oligodendrocyte apoptosis in the central nervous system (CNS). Here, when encephalitogenic T cells are present in the periphery in both autoimmune and viral models of MS, induction of WD in the CNS has been shown to result in the recruitment of T cells along the degenerated tract, leading to demyelination (Inside-Out model). These experimental findings are consistent with early MS pathology described by both “microglial nodules” and “newly forming lesions”.

**Conclusions:**

The differences between the two neuropathological findings may be based on the preference of staining methods, where one group observed axonal and microglial pathology and the other observed oligodendrocyte apoptosis; a Janus face that is looked at from the two different sides.

## Background

Although the precise mechanism of initial lesion development in multiple sclerosis (MS) remains unclear, two different neuropathological findings have been reported as a potential early event of MS: “microglial nodules (MGNs)” and “newly forming lesions (NFLs)” both of which contain neither T cell infiltration nor demyelination [1, 2]. Singh et al. [1] described that damaged axons were associated with a small number of aggregated macrophages/microglia, i.e., MGNs, in the normal appearing-white matter (NAWM) of MS. Microglia expressed major histocompatibility complex (MHC) class II molecules, while axons were immunoreactive to antibodies against non-phosphorylated neurofilament and neuropeptide Y receptor Y1, suggesting that these axons are undergoing Wallerian degeneration (WD). They also found that MGNs were not specific to MS, but also in the affected adjacent NAWM after infarction and traumatic brain injury, but not epilepsy. The authors proposed that MGNs are an early event of MS and occur as a reaction to WD, since the author group has shown that there was no association with blood vessels, blood-brain barrier (BBB) breakdown, or T cell infiltration [1, 3]. The authors discussed how MGNs are different from two other analogous MS lesions: 1) Pattern III lesions by Lucchinetti et al. [4] and 2) NFLs (or prephagocytic areas) [2], since both of which contain oligodendrocyte apoptosis and are described in terms of oligodendrogliopathy. NFLs have been proposed by Barnett et al. [2] as a potential early MS lesion, which are composed of oligodendrocyte apoptosis and microglial activation in NAWM but little or no lymphocytes.

However, is the presence of “MGNs” and “oligodendrogliopathy” mutually exclusive? Might these two different observations be the same neuropathology, but interpreted differently based on the different theories of early MS lesion development, using different staining methods? While MGNs seemed to be initiated by WD, could it be accompanied by oligodendrocyte apoptosis? To address these questions, we discussed that “MGNs” are similar to findings in a viral model of MS, and compared “MGNs” with other early MS lesions, “NFLs”.

## Discussion

### Interaction between axons and oligodendrocytes

Although WD has been demonstrated to induce oligodendrocyte apoptosis along the degenerated tract, for example, after spinal cord injury [5, 6], Singh et al. [1] did not investigate whether oligodendrocyte apoptosis was present in the NAWM. Since oligodendrocyte apoptosis can be induced by WD that disrupts the cross-talk between axons and myelin [7], oligodendrocyte apoptosis can be not only seen in the oligodendrogliopathy whose primary target is oligodendrocytes, but also triggered by primary WD. Clinically and experimentally, it has been shown that oligodendrocyte damage/survival and axonal damage/preservation can influence each other. Which comes first has to be interpreted with caution. It should be noted that neither Lucchinetti's pattern criteria nor Barnett’s NFLs included a detailed evaluation of axonal degeneration, while Barnett et al. [2] described that “axonal changes in general were *relatively* inconspicuous in NFLs of acute MS cases” (the data were not shown). Here, if ‘relatively’ means that a small number of damaged axons were present, their NFLs are similar to MGNs, since microglial activation in the absence of lymphocytes is another characteristic of NFLs [8]. Interestingly, in NFLs, Barnett et al. [2] found complement activation, which has also been demonstrated along the tract of WD [9]; activated complement components within these areas may recruit inflammatory cells into the central nervous system (CNS).

### Outside-In and Inside-Out models in MS

We have proposed two model theories of lesion development in MS: the Outside-In model and the Inside-Out model [5, 10]. In the Outside-In model, MS lesions develop from the outside (myelin) to the inside (axons); in the Inside-Out model, the lesions develop from the inside (axons) to the outside (myelin) [11]. The Outside-In model refers to a primary CNS demyelination, usually induced by anti-myelin autoimmune cells generated in the periphery, while the Inside-Out model refers to a primary CNS axonal degeneration and subsequent recruitment of systemic/adaptive immune cells. The target of effector cells in an autoimmune model of MS, experimental autoimmune encephalomyelitis (EAE), is a myelin antigen; CNS damage in EAE is explained by the Outside-In model. On the other hand, in a viral model of MS, Theiler’s murine encephalomyelitis virus (TMEV) infection, axonal degeneration precedes demyelination [12, 13]; initial lesion development in TMEV-induced demyelinating disease (TMEV-IDD) is explained by the Inside-Out model.

### TMEV-IDD (Inside-Out model) connects MGNs and NFLs

**Fig. 1:**
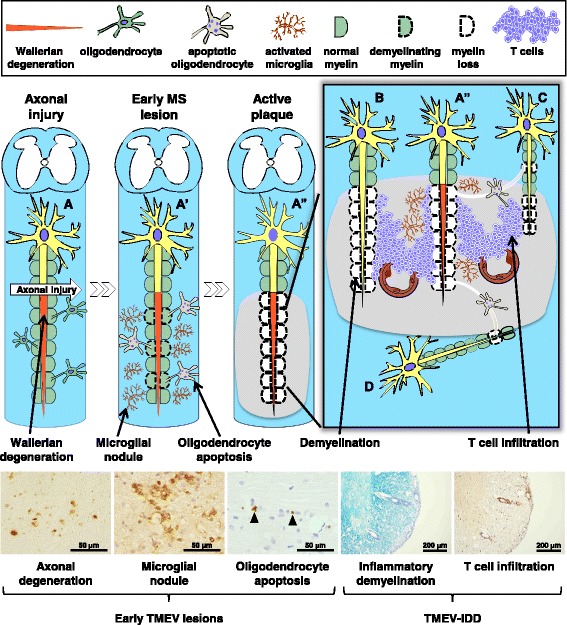
Early lesion development theories of multiple sclerosis (MS) and its viral model. When axonal injury (arrow) occurs in Neuron A, it causes degeneration of the distal part of transected axons [Wallerian degeneration (WD)]. This leads to activation of microglia [microglial nodules (MGNs)] and oligodendrocyte apoptosis along the degenerated axon of Neuron A’, which is followed by demyelination of the nerve fiber of Neuron A”. Here, the lesion develops from the inside axon to the outside myelin (Inside-Out model). Later, the changes in the microenvironment of Neuron A” recruit encephalitogenic T cells from the systemic circulation to the site of WD, where extravasated T cells from the blood vessels attack myelin sheaths of neighboring Neurons B and C, resulting in demyelination. Apoptotic oligodendrocytes that make myelin sheaths of Neuron A” also result in demyelination of Neurons C and D. In this active MS plaque, demyelination of Neurons B, C, and D can occur without the damage of axons (primary demyelination, Outside-In model). In a viral model of MS, Theiler’s murine encephalomyelitis virus (TMEV) infection, axonal degeneration, MGNs, and oligodendrocyte apoptosis are visualized by non-phosphorylated neurofilament staining, lectin cytochemistry [*Ricinus communis* agglutinin (RCA) I], and the terminal deoxynucleotidyl-transferase-mediated dUTP-biotin nick-end labeling (TUNEL) method, respectively, during the early stage of TMEV infection, 1–2 weeks post infection (p.i.), in the normal appearing white matter, preceding demyelination. Full-blown demyelination and T cell infiltration are visualized by Luxol fast blue staining and anti-CD3 immunohistochemistry during the chronic stage of TMEV infection more than 1 month p.i, which is called TMEV-induced demyelinating disease (TMEV-IDD)

In TMEV infection, a small number of damaged axons are detectable in the NAWM of the spinal cord in the absence of demyelination, T cell infiltration, or viral antigen as early as 1 week post infection (p.i.) [10]. Two to three weeks p.i., MGNs are observed along with an increased number of damaged axons in the NAWM (Fig. 1), where activated microglia change from dendritic morphology to more rounded phagocytes, indicating that damaged axons are being phagocytosed. Apoptosis of uninfected oligodendrocytes is also detectable in the NAWM of the spinal cord at this early stage [12], while T cell infiltration and demyelination become obvious after 4 weeks p.i. (chronic stage). The viral antigen is seen in neurons of the gray matter of the brain during the early stage, but in macrophages and glial cells of the white matter of the spinal cord during the chronic stage [10]. Thus, during the early stage of TMEV infection, MGNs, axonal degeneration, and oligodendrocyte apoptosis are present in the NAWM of the spinal cord, which precedes demyelination and T cell infiltration. This pathology is similar to the findings regarding early MS lesions described by *both* the Singh and the Barnett groups. Interestingly, one of the characteristics of TMEV-IDD is vacuolar demyelination, in which lesions appear vacuolar because of axonal swelling. Although Barnett et al. [2] also described that the NFLs appeared “vacuolar”, the authors speculated that the cause of the vacuolated tissue was because of the presence of widespread intramyelinic edema.

Induction of MHC class II molecules in microglial activation (i.e., MGNs) along the tract of WD was first demonstrated experimentally by Konno et al. [14]. Then, encephalitogenic T cells were adoptively transferred to induce passive EAE in rats, in which MHC class II molecules had been induced on microglia at the sites of WD without BBB breakdown. The recipient rats developed distinct inflammatory lesions that corresponded with the distribution of activated microglia; e.g., in the ipsilateral thalamus after cortical cryoinjury [15]. Here, axonal degeneration in the presence of MGNs recruited encephalitogenic T cells from the periphery to the sites of WD. We confirmed this in TMEV-IDD [16]. We induced WD without BBB breakdown in the one side of the dorsal funiculus of the spinal cord by killing dorsal root ganglion (DRG) cells by toxic lectin injection into the one side of sciatic nerve. Lectin injection alone induced WD with MGNs in the ipsilateral half of the dorsal funiculus with no inflammatory demyelination. If the same procedure was conducted in TMEV-infected mice, T cell infiltration and demyelination were induced along the sites of the WD. Since the dorsal funiculus is spared in control mice with TMEV-IDD, experimentally-induced WD with MGNs likely recruited T cells to the sites of WD. These findings showed that 1) WD alone is enough to induce MGNs (but not demyelination), 2) it can also recruit encephalitogenic T cells, only if these T cells are present in the periphery, and 3) these T cells are required for full blown demyelination. This may explain why there have been only anecdotal reports that CNS neurotrauma was associated with the onset of MS; for recruitment of T cells into the CNS, the presence of activated T cells in the periphery that have a potential to home CNS is required (such as T cells specific for CNS antigens or T cells specific for viruses that persistently infect the CNS).

### The term, “preactive lesions (PALs)”, includes MGNs, NFLs, and other early MS lesion concepts

As we discussed above, experimentally, the lesions with histological features of both MGNs and NFLs can be reproduced in animal models of MS. In human MS, Amor and her collaborating groups (e.g., van der Valk and van Noort) have used the term, “preactive lesions (PALs)”, which link seemingly unrelated findings and theories of early MS lesions, based on by their own findings as well as neuropathology and neuroimaging findings by others [17–20]. The concept “PALs” includes MGNs, NFLs, Pattern III MS lesions, and other early MS lesions, based on the common pathological findings: microglial activation in the apparent absence of infiltrating leukocytes before any signs of apparent demyelination. In “PALs”, activated microglia co-clustered with apoptotic oligodendrocytes and/or oligodendrocytes expressing anti-apoptotic molecules, suggesting that oligodendrocyte stress may trigger microglial activations. Other possible triggers for microglial activation in PALs include axonal degeneration and infections [17, 19]; induction of pattern recognition receptors, such as toll-like receptors (TLRs), has been shown in PALs [21, 22]. Neuroimaging studies of NAWM are consistent with the concept of PALs [17].

While the current concept of “PALs” seems to reasonably unify most early MS lesion concepts, there have been changes in the usages of terminology of early MS lesions (depending on the authors), which may hamper neuropathologists’ ability to reach a consensus on early MS lesion pathology. One issue is the presence of perivascular lymphocyte infiltration. The current concepts of PALs and MGNs as well as NFLs in the original article agree that microglial activation occurs without perivascular lymphocyte extravasation into CNS parenchyma in early MS lesions. Confusingly, however, mild perivascular lymphocyte infiltration has been seen occasionally in several pathology manuscripts on early MS lesions: for example, 1) the first manuscripts that described “(p)reactive lesion” by De Groot et al. [23] defined PALs as “perivascular accumulation of lymphocytes in microvessels”, and 2) more recent manuscripts on “NFLs” described mild T cell infiltration [24]. In addition, oligodendrocyte apoptosis and axonal degeneration have been rarely examined simultaneously, while the presence of axonal degeneration in early MS lesions differed among the reports: e.g. detected (or correlated) [1], not detected (not correlated) [3], or not examined by most reports.

### Remaining questions: 1) Transition from early MS lesions to active MS plaques and 2) Role of astrocytes

PALs have been suggested not necessarily leading to lymphocyte infiltration; PALs may not always develop into inflammatory demyelinating lesions but can resolve without subsequent disorder [19]. Although the precise molecular mechanism that determines the development from PALs to inflammatory demyelinating lesions is not clear in MS or its animal models, it has been proposed that production of anti-inflammatory cytokines, such as interleukin (IL)-10, from microglia would lead to lesion resolution. On the other hand, induction of pro-inflammatory cytokines, such as tumor necrosis factor (TNF)-α, from activated microglia likely activates endothelial cells [18, 19], leading to upregulation of adhesion molecules on endothelial cells, including vascular cell adhesion molecule (VCAM)-1 and intracellular adhesion molecule (ICAM)-1 [25]. Elucidation of these plausible sequential changes in cytokine and adhesion molecule profiles will lead to better understanding of a transition from early MS lesions to active MS plaques.

Lastly, although we discussed the involvement of three major parenchymal cell types of the CNS, microglia, neurons (axons), and oligodendrocytes, in early MS lesions, the role of the forth major cell type, astrocytes, remains unclear. Although only a few manuscripts briefly described the absence of astrogilosis and a lack of upregulation of MHC molecules on astrocytes in early MS lesions [3, 17], quantitative and time course analyses of astrocytes have not been a major focus of early MS lesion studies both clinically and experimentally, including the TMEV model. However, regardless of being activated or not, astrocytes could play a role in the early MS lesions. For example, astrocytes are a component of neurovascular and gliovascular units, interacting with blood vessels, and regulating blood flow and BBB functions [26]. Axonal damage, not only in the CNS but also peripheral nerves (e.g., induced by peripheral nerve ligation or toxin injection), has been shown to induce astrocyte activation, while peripheral nerve injury can also activate satellite glial cells in the dorsal root ganglion [27, 28]. Such activated astrocytes (and satellite glial cells) have been shown to produce pro-inflammatory cytokines, such as TNF-α and IL-1β, which can impair BBB function [16, 26]. Thus, the changes in astrocytes are worth examining together with microglia, axonal, and oligodendrocyte pathology in early MS lesions and its animal models.

## Conclusions

WD has been shown to induce MGNs (microglial activation) and oligodendrocyte apoptosis. WD can result in the recruitment of T cells along the degenerated tract, leading to demyelination in both autoimmune and viral models. These experimental findings are consistent with early MS pathology described by *both* the Singh and the Barnett groups, and support that MGNs and NFLs can be an early MS lesion. The differences between the two research groups may be based on their theories and staining methods; a Janus face that is looked at from the two different sides.

## Abbreviations

BBB: Blood-brain barrier
CNS: Central nervous system
EAE: Experimental autoimmune encephalomyelitis
IL: Interleukin
MGNs: Microglial nodules
MHC: Major histocompatibility complex
MS: Multiple sclerosis
NAWM: Normal appearing-white matter
NFLs: Newly forming lesions
PALs: Preactive lesions
p.i.: Post infection
TMEV: Theiler’s murine encephalomyelitis virus
TMEV-IDD: TMEV-induced demyelinating disease
TNF: Tumor necrosis factor
WD: Wallerian degeneration
